# Hybrid fragment-SMILES tokenization for ADMET prediction in drug discovery

**DOI:** 10.1186/s12859-024-05861-z

**Published:** 2024-08-01

**Authors:** Nicholas Aksamit, Alain Tchagang, Yifeng Li, Beatrice Ombuki-Berman

**Affiliations:** 1 Department of Computer Science, Brock University, 1812 Sir Isaac Brock Way, St. Catharines, ON L2S 3A1 Canada; 2 Digital Technologies Research Centre, National Research Council Canada, 1200 Montreal Road, Ottawa, ON K1A 0R6 Canada; 3 Department of Biological Sciences, Brock University, 1812 Sir Isaac Brock Way, St. Catharines, ON L2S 3A1 Canada

**Keywords:** ADMET prediction, Transformer, Fragments, SMILES, Drug discovery

## Abstract

**Background:**

Drug discovery and development is the extremely costly and time-consuming process of identifying new molecules that can interact with a biomarker target to interrupt the disease pathway of interest. In addition to binding the target, a drug candidate needs to satisfy multiple properties affecting absorption, distribution, metabolism, excretion, and toxicity (ADMET). Artificial intelligence approaches provide an opportunity to improve each step of the drug discovery and development process, in which the first question faced by us is how a molecule can be informatively represented such that the in-silico solutions are optimized.

**Results:**

This study introduces a novel hybrid SMILES-fragment tokenization method, coupled with two pre-training strategies, utilizing a Transformer-based model. We investigate the efficacy of hybrid tokenization in improving the performance of ADMET prediction tasks. Our approach leverages MTL-BERT, an encoder-only Transformer model that achieves state-of-the-art ADMET predictions, and contrasts the standard SMILES tokenization with our hybrid method across a spectrum of fragment library cutoffs.

**Conclusion:**

The findings reveal that while an excess of fragments can impede performance, using hybrid tokenization with high frequency fragments enhances results beyond the base SMILES tokenization. This advancement underscores the potential of integrating fragment- and character-level molecular features within the training of Transformer models for ADMET property prediction.

## Introduction

Drug design has evolved from serendipitous screening of natural compounds to an increasingly rational and data-driven approach, focusing on the molecular structure and mechanisms behind disease-related targets [1]. The application of artificial intelligence (AI), particularly machine learning (ML), has revolutionized the pharmaceutical field, which is able to take advantage of the vast arrays of biomedical data that has been gathered [2]. AI and ML contribute to various aspects of drug design, including predicting pharmacokinetic and pharmacodynamic properties, identifying binding sites on a given biomolecular target, repurposing drugs, and creating new molecules with desired characteristics, all of which reduce the time and cost associated with developing effective and safe medications [3–5]. Furthermore, absorption, distribution, metabolism, excretion, and toxicity (ADMET) are crucial in evaluating drug post-administration behaviour, and in minimizing clinical trial failures [6, 7]. Despite challenges, such as data scarcity and complex molecular structures in the area of ADMET prediction, ML techniques have been able to extrapolate structural patterns that implicate molecular properties, and circumvent the need for costly assays during a large-scale screening process. As a result, ML plays a significant role in the identification and early exclusion of unsuitable compounds, mitigating financial burdens from unsuccessful ventures in the drug development cycle.

In the field of computational chemistry, molecular structures can be represented through various formats. Line notations, such as Simplified Molecular Input Line Entry System (SMILES) [8] provide a textual method to describe the structure of chemical entities, including molecular information such as C for carbon, = for a double bond, parentheses for branches, and @, /, /, for stereochemistry. As an example, climbazole is represented as CC(C)(C)C(=O)C(N1C=CN=C1)OC2=CC=C(C=C2)Cl. Despite the widespread use of SMILES, its strict syntactical guidelines often result in the production of numerous invalid molecular structures, leading to the development of other line notations such as DeepSMILES [9], SELF-Referencing Embedded String (SELFIES) [10], and Group SELFIES [11] which mitigate some of the mentioned issues. However, they are not as widely supported as SMILES and may necessitate larger alphabets.

Fragmentation is another approach to representing a molecule where a large molecule is broken apart into smaller pieces [12]. The fragmentation process can reveal important structural and functional features of the original molecule that are not easily discernible from an atomic-level representation such as SMILES. For example, fragmentation can generate sub-molecules that contain specific functional groups or motifs that are relevant for physicochemical properties. However, fragmentation is complex due to the variety of methods and criteria involved in bond cleavage and the selection of sub-molecular entities [13]. Additionally, fragmentation presents several challenges, such as producing sub-molecules that are unusually large or small, and the formation of a vast library of fragments that appear with varying frequencies, with a significant number rarely occurring.

**Fig. 1:**
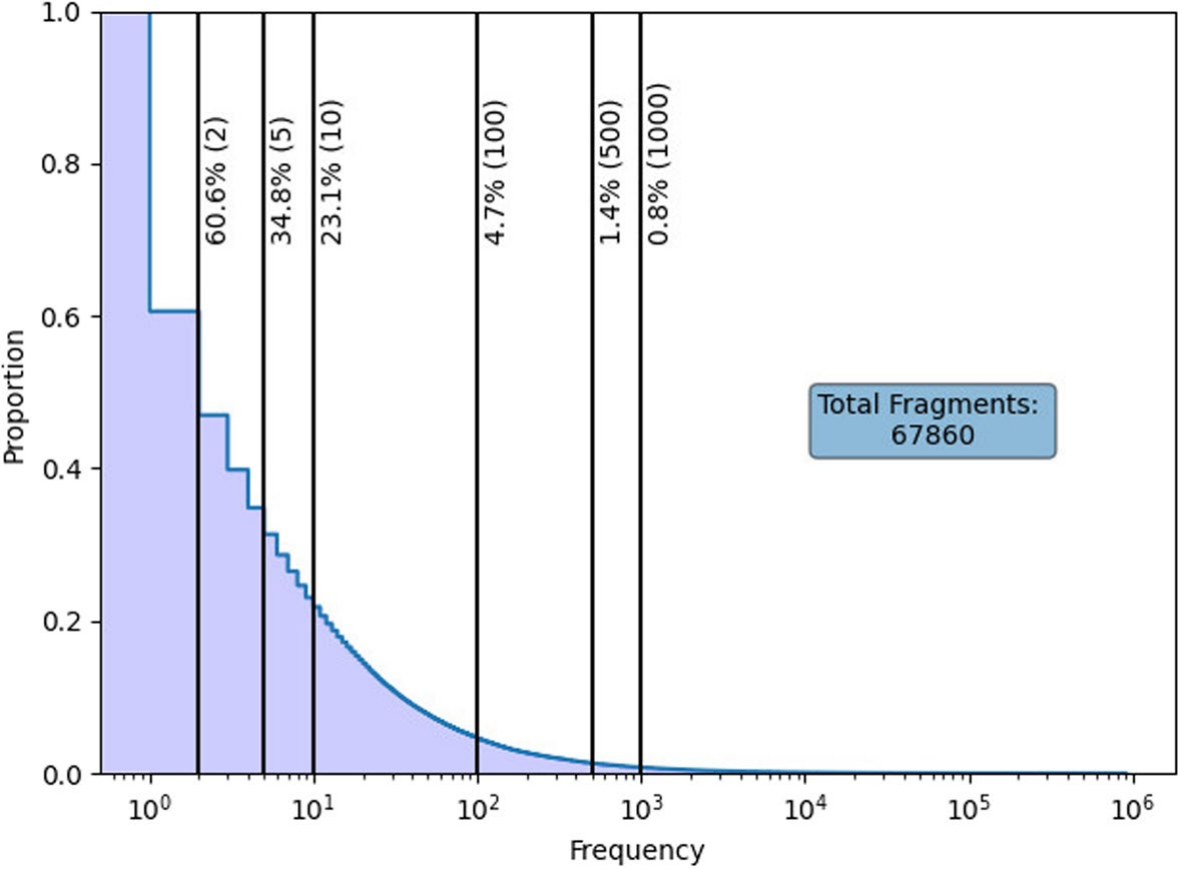
Fragment library proportions by observation frequency. An integer in the brackets is a threshold. The corresponding percentage represents the portion of fragments above the threshold

To the best of our knowledge, there exists no direct comparison between fragment and atom-based models for ADMET prediction using the same model. In this study, we construct a hybrid fragment-SMILES encoding technique to combine the advantages of both representations for use in machine language models. As illustrated in Fig. 1, there are a large amount of fragments that occur infrequently. Thus, we construct various models with varying frequency cutoffs to produce a fragment spectrum of models, and perform a fair comparative investigation between SMILES and a fragment spectrum using the hybrid encoding technique for ADMET prediction with a Transformer architecture. Moreover, we also experiment with two pre-training techniques which we denote one-phase and two-phase.

The rest of this study is organized as follows. Section "Related works" discusses related works, giving information on the use of Transformers for ADMET prediction, and graph-based neural networks for ADMET prediction. Section "Methodology" discusses the methods used within this work, describing the model and the encoding approach. Afterwards, Section "Experimentation" illustrates all the necessary information for replicating the experiments performed in this study. Following is Section "Results and discussions" which displays and investigates the results of our experimentation. Lastly, concluding remarks are made in Section "Conclusion".

## Related works

### Transformer-based ADMET models

Language models are a class of deep learning models that learn the semantic and syntactic patterns of natural language from a large corpora of text. They can also be applied to molecular sequences, such as SMILES strings, to capture the structural and functional features of molecules. Before the advent of Transformer models, recurrent neural networks (RNNs) were commonly used for language modelling tasks, and also for tasks within ADMET prediction [14, 15]. One of the advantages of language models is their ability to leverage pre-trained weights in an unsupervised or semi-supervised fashion over a general domain, and then fine-tune for downstream tasks such as ADMET prediction. This process, known as transfer learning, helps improve performance and generalization capabilities by reducing the risk of overfitting and increasing the diversity of molecules for syntactic and semantic understanding. Transfer learning is particularly useful when the data is scarce or noisy, such as is frequent in ADMET prediction where gathering data is costly.

Attention mechanisms are not novel, as they have been applied in RNNs before the birth of Transformers [16, 17], however the Transformer architecture emphasizes self-attention to focus on the important sequence sections and capture long-range dependencies and relationships among them [18]. As Transformers have shown superior performance over RNNs for natural language processing (NLP) tasks, they too have become a focus of research towards ADMET prediction. Many of the constructed ADMET Transformer models make use of those popularized in NLP literature, such as but not limited to BERT, RoBERTa, and GPT-2 [19–29]. Others combine graph representations with the Transformer to obtain graph-level contextual understanding [30–36]. In addition, some works use a combination of molecular line notation and pre-fabricated descriptors [25], while the remaining use the Transformer with various training strategies and architectural changes [37–40]. Despite the application of various modelling strategies, including pre-training techniques and attention mechanisms for ADMET prediction, a common thread in prior research is the use of SMILES or molecular graph representations. This differs from our investigation which utilizes a hybridized fragment and SMILES encoding using the MTL-BERT model [41].

Although Transformer models have been proposed for ADMET prediction, they face several challenges and limitations, such as data availability, data quality, model interpretability, and robustness. Both data availability and quality are crucial for training accurate and reliable ADMET prediction models, however many ADMET datasets are class imbalanced and can at times be inconsistent. In addition, datasets are often imbalanced in terms of sample quantity when combined for training purposes, and improper sampling strategies are likely to cause catastrophic forgetting among tasks when considering a multi-task approach [42]. Model interpretability and robustness are important for understanding the rationale behind predictions and ensuring their applicability in varying scenarios. Equally as important, Transformer models must also be computationally efficient and scalable to handle large-scale and complex molecular datasets. This becomes important with the rise of foundational models in NLP literature, which has in turn spilled over into the cheminformatics domain [37].

### Graph-based ADMET models

Graph-based neural network (GNN) models are a popular and effective way of leveraging information gained from using graph-structured data such as molecules [43]. A graph is a collection of nodes and edges, where nodes are the building blocks, such as atoms, and edges are connections between entities, like bonds. GNNs learn meaningful representations of molecular structures and properties by aggregating information from local neighbourhoods through different operations such as message passing or convolution. Attention may also be included in GNNs to determine the important neighbouring node features to aggregate. Afterwards, node features constructed by the model can then be combined to obtain a feature vector for the whole graph, which is in turn used for downstream tasks such as ADMET prediction. GNNs have been widely used in ADMET prediction as they can capture the structural and chemical properties of molecules similar to SMILES, and similarly to Transformer models, have the ability to leverage structural information of molecules through transfer learning [44, 45].

GNNs applied to ADMET prediction can be broadly categorized into four groups: graph convolutional neural networks (GCNs) [43], graph attention networks (GATs) [46], message passing neural networks (MPNNs) [47], and graph isomorphism networks (GINs) [48]. GCNs apply convolution operations on the graph nodes to learn node embeddings, which are then pooled to obtain a graph-level representation. MT-PotentialNet [49], Weave [50], and other models [51, 52], are notable works that fall into this division. GATs use attention mechanisms to assign varying weights to neighbouring nodes and edges, allowing the model to focus on the most relevant parts of the graph. ADMETLab 2.0 [53], AttentiveFP [54], and GASA [55] are examples of GATs for ADMET prediction. MPNNs use a message passing scheme to propagate information across the graph, where each node sends and receives messages from its neighbours, and then updates its own hidden state accordingly. In this regard there are models like D-MPNN [56], GeomGCL [57], and MGSSL [58]. Lastly, GINs generalize the Weisfeiler-Lehman graph isomorphism test and learn node embeddings by aggregating and transforming features of neighbouring nodes with learnable parameters, afterwards pooled for a graph-level representation. This powerful GNN variant is seen represented in MolGIN [59]. It should also be noted that many GNN models for ADMET prediction use a hybridization of the various categories to improve performance [60–64], including multi-task learning to leverage information from multiple ADMET datasets, some of which may have a low amount of samples. As is discussed in [65], many works using graph-based neural network models consider 2D chemical topology, but disregard geometrical data that provides useful information when predicting molecular properties.

## Methodology

### Hybrid fragment-SMILES tokenization

Prior to inputting a molecule into a Transformer model, it must undergo tokenization and be encoded into a numerical representation. We propose a novel tokenization procedure, named hybrid fragment-SMILES tokenization (HFST), that incorporates both fragments and SMILES, where SMILES fragments are generated using the method described in HierVAE [66] and DeepFMPO [12]. Following their technique, bonds connected away from a ring atom are broken, and an attachment point is inserted for later molecule reconstruction. To encode a molecule using the hybrid method, we first fragment and loop through all fragments. If a fragment is in the vocabulary, we use a single numerical value for encoding. Otherwise, we encode the fragment using the SMILES atomic-level representation. If two or more successive fragments are encoded using SMILES, a separator tag is placed in-between them to designate the ending of one fragment and the start of another.

**Fig. 2:**
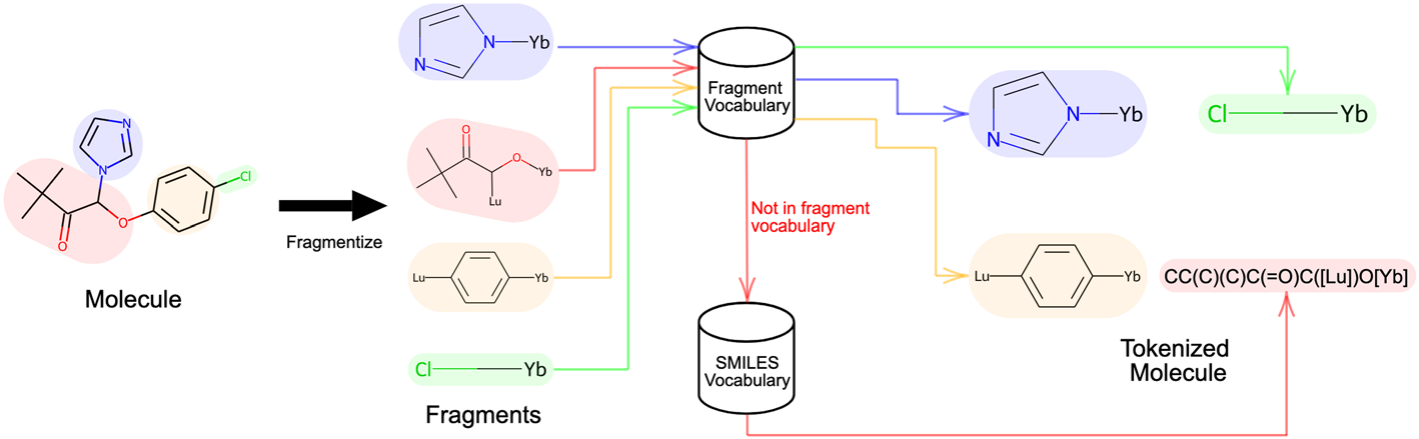
Illustration of the hybrid SMILES-fragment encoding process using climbazole (SMILES representation: CC(C)(C)C(=O)C(N1C=CN=C1)OC2=CC=C(C=C2)Cl)

The advantages of using the HFST encoding over the standard SMILES encoding are fourfold. (1) It overcomes the issue of low-frequent fragments in training. From Fig. 1, we can see that the frequency of fragments follows a power-low distribution. Low-frequent fragments dominate the over-sized vocabulary and thus lead to poor contextual embedding. (2) It solves the problem of new fragments in inference. In predictive tasks, some fragments of new molecules may not exist in the vocabulary of fragments extracted from the training data. Using an unknown token leads to information missing. (3) The fragment spectrum perspective unifies fragment-based and SMILES-based tokenizations, allowing us to select a cutoff that takes benefits of both fragment and SMILES representations. Fragment-based (no cutoff) and SMILES (all cutoff) are extreme cases of the HFST representation. (4) It can reduce the sequence length and thus reduce some computational complexity of the Transformer model, which is quadratic with length of input sequence. Our HFST method is illustrated in Fig. 2, where a singular molecule on the left is first fragmented, as seen by the colours blue, red, yellow, and green. Afterwards during encoding, the fragments shaded as blue, yellow and green were found in the vocabulary and so take the form of a single numerical value. However, the red fragment is encoded as a SMILES string as it was not found in the vocabulary, and thus will be encoded as many numerical values, one for each SMILES token.

### Transformer ADMET model

**Fig. 3:**
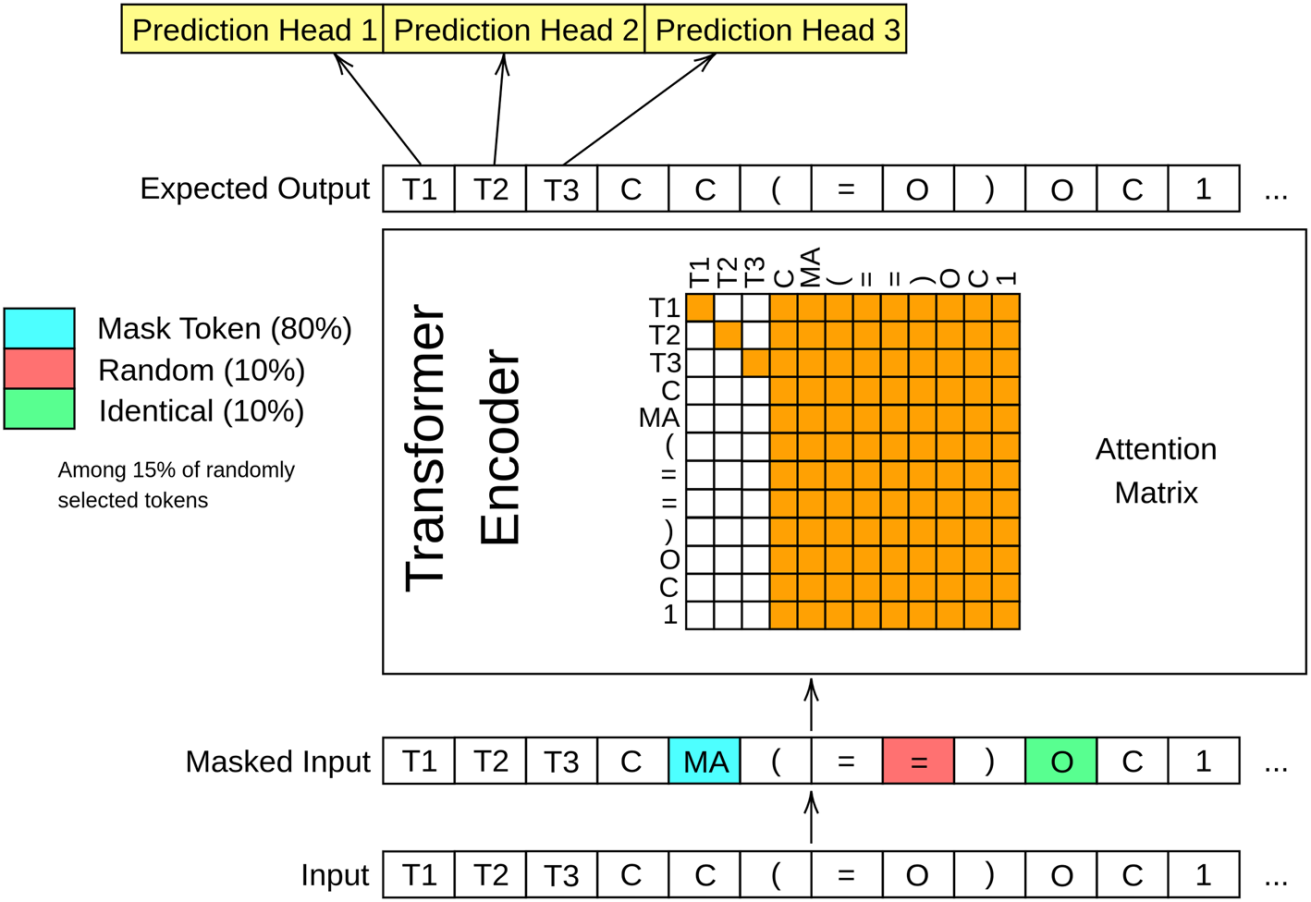
Schematic illustration of the MTL-BERT model architecture that utilizes the Transformer encoder, incorporates masked language modelling for pre-training, and operates with multiple task-specific tokens and heads in fine-tuning

We adopt the MTL-BERT model originally proposed by Zhang et al. for predicting ADMET properties from SMILES strings [41]. The original MTL-BERT is depicted in Fig. 3. It uses a multi-task learning framework based on BERT, a Transformer-based model that learns bidirectional representations from large-scale unlabelled data. Similarly to BERT, MTL-BERT uses transfer learning which consists of two parts. In the pre-training phase, a masked language model objective is used to learn the contextual information of SMILES sequences from a large corpus of unlabelled molecules. Unlike the BERT model, next sequence prediction is not included as a pre-training objective. Afterwards, the pre-trained MTL-BERT model is fine-tuned on multiple downstream prediction tasks simultaneously with the inclusion of multiple task-specific tokens and prediction layers. The prepending of multiple task-specific tokens to a sequence, one for each predictive task, differs from the original BERT model which prepends a singular token. In the original work by Zhang et al., SMILES enumeration was used as a data augmentation technique to increase diversity, however this is not included in our work due to the generation of rare fragments not present in our curated library.

MTL-BERT is selected as the model for this study as it leverages large-scale unlabelled data to learn contextual information about SMILES strings during pre-training, which has been shown in previously mentioned studies to improve performance in downstream tasks. In addition, as MTL-BERT is inherently a multi-task model, it can benefit from sharing information amongst multiple tasks, enhancing the generalization capability of the model. Multi-task learning is particularly useful for ADMET prediction as there are numerous predictive tasks, many of which have a low amount of samples. Furthermore, as reported in [41], MTL-BERT outperforms the multi-task graph attention (MGA) framework [53] for the same ADMET tasks. We were unable to set up the MGA framework because the code from https://github.com/wzxxxx/MGA is incomplete and lacks of important details. However, we believe that adopting MTL-BERT for the HFST representation in our study is sufficient due to MTL-BERT’s reported superior performance.

## Experimentation

This section outlines our experimental procedures to assess the performance of the proposed HFST method for ADMET prediction. First, we describe the data used for training our models. This is followed by examining the alteration of fragment vocabulary size through various frequency thresholds, resulting in a spectrum of fragments. Afterwards, the hyperparameters utilized during training to ensure replicability are presented. Additionally, two strategies for pre-training the Transformer model on large-scale unlabelled data are introduced. Lastly, the description of the metrics and methodologies used to ensure an equitable evaluation of the models is given.

### Data and preprocessing

We train our model with transfer learning, which segregates the training process into two parts: pre-training and fine-tuning. During the pre-training stage, we use a large collection of unlabelled molecules to train our Transformer models. This ensures the model acquires a generalized representation of molecular structures through self-supervised learning, specifically with masked language modelling. The pre-training data consists of molecules from ChEMBL [67], MOSES [68], and ZINC-250K [14] datasets, where canonical duplicates are removed and SMILES strings above 100 tokens discarded. In total, the dataset comprises roughly 4 million molecules, which is divided into a random 80-20 train-test split.

In the fine-tuning stage, a variety of smaller datasets are utilized to fine-tune the pre-trained network, enabling it to concurrently predict 29 ADMET properties through multi-task learning. The fine-tuning data consists of molecules with experimentally measured ADMET values from different sources, having a combined size of 108,315 samples. In line with our pre-training data, SMILES sequences that surpass 100 tokens are removed. Table 1 illustrates the various ADMET datasets with their accompanying size, task type, and ADMET category. All ADMET datasets for fine-tuning were obtained from Therapeutics Data Commons (TDC) [69].

**Table 1:** Fine-tuning datasets

ADMET	Dataset name	Size	Type
Absorption	Caco-2	824	Regression
Absorption	PAMPA permeability	1725+/286−	Classification
Absorption	HIA	493+/59−	Classification
Absorption	Pgp inhibition	631+/547−	Classification
Absorption	Bioavailability	478+/127−	Classification
Absorption	Lipophilicity	4189	Regression
Absorption	Solubility	9757	Regression
Absorption	FreeSolv	642	Regression
Distribution	BBB	1521+/411−	Classification
Distribution	PPBR	1600	Regression
Distribution	VDss	1036	Regression
Metabolism	CYP 2C19	5783+/6625−	Classification
Metabolism	CYP 2D6	2491+/10379−	Classification
Metabolism	CYP 3A4	5036+/7055−	Classification
Metabolism	CYP 1A2	5822+/6502−	Classification
Metabolism	CYP 2C9	4012+/7817−	Classification
Metabolism	CYP2C9 substrate	140+/493−	Classification
Metabolism	CYP2D6 substrate	189+/442−	Classification
Metabolism	CYP3A4 substrate	330+/302−	Classification
Excretion	Half life	591	Regression
Excretion	Hepatocyte clearance	1196	Regression
Excretion	Microsome clearance	1099	Regression
Toxicity	LD50	7362	Regression
Toxicity	hERG	443+/195−	Classification
Toxicity	AMES	3961+/3289−	Classification
Toxicity	DILI	228+/232−	Classification
Toxicity	Skin reaction	274+/130−	Classification
Toxicity	Carcinogens	51+/188−	Classification
Toxicity	ClinTox	100+/1232−	Classification

### Fragment spectrum

To apply the hybrid fragment-SMILES encoding of molecules, we constructed two vocabularies: one for SMILES and one for fragments. These vocabularies are derived exclusively from the pre-processed pre-training data, as described in the previous section. We use the RDKit Python package [70] to tokenize SMILES strings and the fragmentation technique from HierVAE [66] and DeepFMPO [12] to generate the fragments. Figure 1 illustrates the proportional distribution of fragment frequencies within the pre-training dataset, highlighting that a majority of fragments are uncommon, with only a select few being prevalent. The figure further segments the cutoff thresholds with vertical lines and shows the proportion of fragments meeting or surpassing these values, emphasizing the rarity of most fragments. In this study, we explore the impact of different fragment frequency thresholds ranging from 2 to 1000, as well as the absence of any threshold, on the efficacy of a Transformer model in predicting ADMET properties using our hybrid tokenization method.

### Model hyperparameters

**Table 2:** MTL-BERT hyperparameters

Name	Value
Pre-train learning rate	1e–4
Fine-tune learning rate	5e–5
Embedding dimension	256
Transformer layers	8
Self-attention heads	8
Feedforward dimension	1024
Dropout	0.1
Batch size	64
Random seeds	[42, 182, 625, 511, 310]

As mentioned previously, we adopt the MTL-BERT model proposed in [41] as the backbone of our experimental framework. In their work, Zhang et al. categorized hyperparameter values by small, medium, and large, where it was reported that the medium parameter size achieved a good balance between predictive performance and computational efficiency. Therefore, we follow their settings and use the medium hyperparameters for our model, as shown in Table 2. Specifically, our model has a hidden size of 256, 8 encoder layers, 8 attention heads, a dropout rate of 0.1, and a feedforward dimension of 1024. We use the Adam optimizer with a learning rate of $1e^{-4}$, betas 0.9 and 0.98, and cross entropy loss to pre-train our model on a large corpus of molecules. Then, we fine-tune our model on the task-specific datasets using the AdamW optimizer with a learning rate of $0.5e^{-4}$, the same beta values as in pre-training, mean squared error loss for regression tasks, and binary cross entropy loss for classification tasks.

For both pre-training and fine-tuning, we set the batch size to 64. To monitor the training progress and avoid overfitting, we conduct a testing epoch every 5000 training batches during pre-training and stop the training process if there is no improvement in the testing loss for two consecutive epochs. For fine-tuning, we perform a testing epoch after every training epoch and terminate if the testing loss increases two epochs in a row. In the pre-training stage, 15% of tokens are chosen at random. Of these, there is an 80% probability that a token is substituted with a mask token, a 10% probability of alteration to a random token, and a 10% probability that it will remain unchanged.

### One-phase and two-phase pre-training

We experiment with two different pre-training strategies for our Transformer model: one-phase and two-phase. In both strategies, we use a large corpus of unlabelled molecular structures as the pre-training data, accompanied with masked language modelling objectives. In one-phase pre-training, the Transformer model is pre-trained using the hybrid fragment-SMILES encoding. This strategy allows the model to directly learn the hybrid encoding without any intermediate steps. In two-phase pre-training, the Transformer model is pre-trained first on the SMILES encoding until no further performance improvement, and then afterwards on hybrid fragment-SMILES encoding until completion. The two-phase approach is designed to capitalize on the insights gained from SMILES encoding before learning the hybrid SMILES-fragment encoding. We hypothesize that the inclusion of low-frequency fragments in the fragment vocabulary may result in reduced visibility of SMILES tokens. Thus, two-phase pre-training allows the model to gradually adapt to the hybrid encoding while preserving the knowledge learned from SMILES embeddings. After pre-training, we perform fine-tuning on the various ADMET datasets using the pre-trained model.

### Evaluation

We evaluate the performance of our Transformer model under three scenarios: pre-training (section Pre-training), fine-tuning on 29 ADMET datasets (section Fine-tuning for ADMET prediction), and fine-tuning on the ADMET group benchmarks from TDC (section Fine-tuning on therapeutics data commons ADMET benchmark). For pre-training, we compare model performance by utilizing testing loss and accuracy. For fine-tuning on the 29 ADMET datasets, we compare using area under the receiver operating characteristic curve (AUROC) on classification tasks, and the coefficient of determination ($R^2$) on regression tasks, both from the testing set, as is indicated in Table 1. For fine-tuning on the ADMET group benchmarks from TDC, various evaluation methods are employed, including mean average error (MAE), AUROC, Spearman’s rank correlation coefficient (Spearman), area under the precision-recall curve (AUPRC), each of which are identified, along with its corresponding dataset, in Table 6. Similarly, we report the testing set performance on benchmark datasets.

Since the combination of ADMET datasets used in this study are imbalanced with sample size, we adopt a stratified batching strategy during fine-tuning on the 29 ADMET datasets and benchmark datasets, ensuring that each batch contains at least one sample from each dataset. By adopting this approach, we prevent the model from overfitting to larger datasets where samples may be overrepresented in batches, thereby enhancing model generalization. To mitigate the impact of data partitioning on model performance variability, we repeat the entire training procedure 5 times using distinct random seeds specified in Table 2. We also implement fivefold cross-validation when fine-tuning with the 29 ADMET datasets and present both the mean and the standard deviation of the performance metrics across all folds and the 5 training runs. For fine-tuning on the TDC benchmark datasets, a 70–10–20 train–validation–test scaffold split [71] is performed, with 5 training runs, along with expression of the mean and standard deviation of all performance metrics.

## Results and discussions

### Pre-training

**Table 3:** Pre-training results on the testing set in terms of mean and standard deviation among five executions

Model	Test loss	Test accuracy
No Cutoff (1P)	$1.022 \pm 0.006$	$0.804 \pm 0.003$
No Cutoff (2P)	$1.030 \pm 0.021$	$0.810 \pm 0.004$
2 Freq. (1P)	$0.999 \pm 0.009$	$0.805 \pm 0.005$
2 Freq. (2P)	$1.017 \pm 0.014$	$0.812 \pm 0.003$
5 Freq. (1P)	$0.969 \pm 0.009$	$0.811 \pm 0.004$
5 Freq. (2P)	$0.997 \pm 0.020$	$0.814 \pm 0.004$
10 Freq. (1P)	$0.936 \pm 0.006$	$0.814 \pm 0.004$
10 Freq. (2P)	$0.948 \pm 0.012$	$0.821 \pm 0.001$
100 Freq. (1P)	$0.739 \pm 0.007$	$0.841 \pm 0.003$
100 Freq. (2P)	$0.765 \pm 0.014$	$0.840 \pm 0.003$
500 Freq. (1P)	$0.551 \pm 0.012$	$0.864 \pm 0.004$
500 Freq. (2P)	$0.554 \pm 0.019$	$0.867 \pm 0.003$
1000 Freq. (1P)	$0.467 \pm 0.012$	$0.876 \pm 0.003$
1000 Freq. (2P)	$0.465 \pm 0.011$	$0.879 \pm 0.002$
SMILES	${0.108} \pm {0.003}$	${0.962} \pm {0.001}$

1P: one-phase pre-training. 2P: two-phase pre-training

**Fig. 4:**
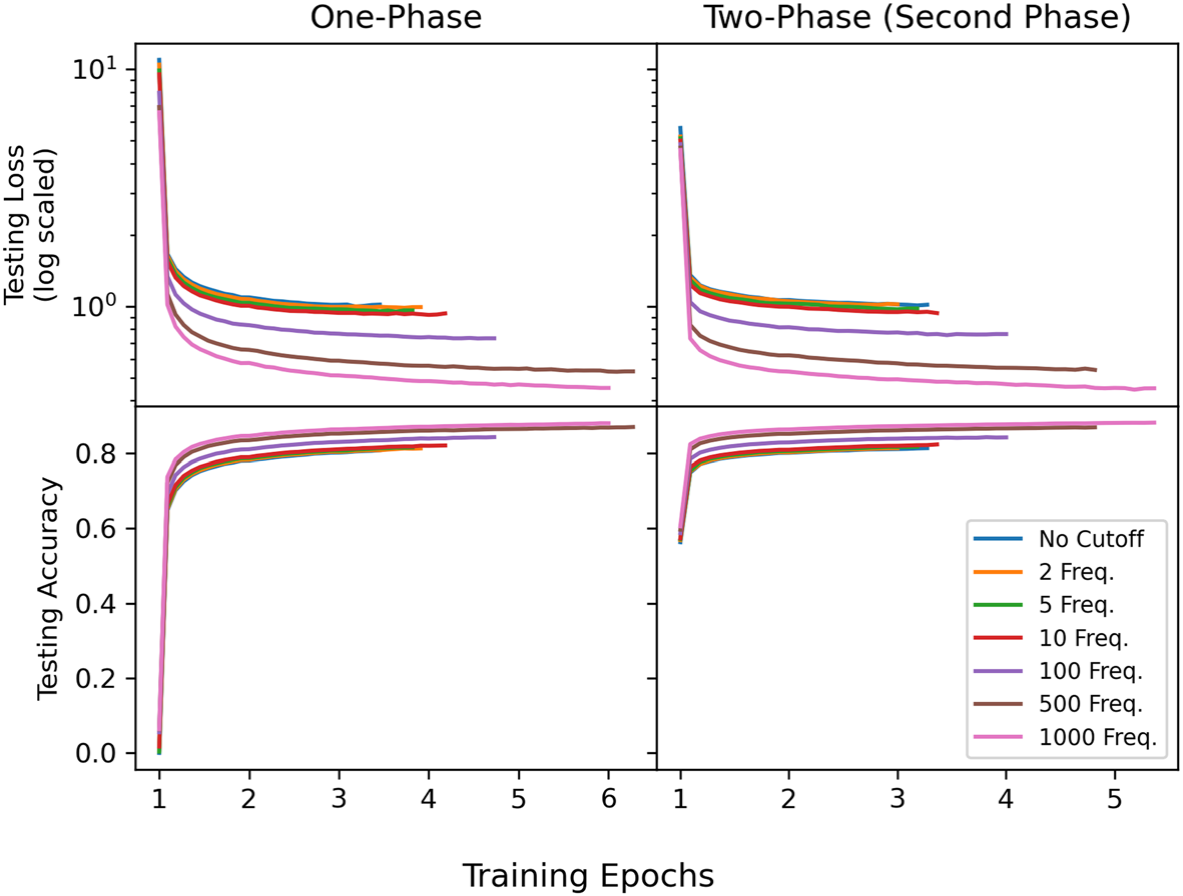
Pre-training curves in one-phase and two-phase strategies, averaged among executions

The results of all pre-training experiments in the final testing epoch, with mean and standard deviation among the five executions, is shown in Table 3. Indicated is a negative correlation between the frequency cutoff and both loss and accuracy. The accuracy in the pre-training stage is calculated for the prediction of masked tokens. This implies that MTL-BERT has more difficulty in predicting the masked fragment tokens when infrequent, and diverse fragments are used in place of SMILES. With a reduction in the cutoff, there is a swift rise in the number of fragments, as depicted in Fig. 1. A decrease in frequency cutoff is likely to lead to lower accuracy due to the increased presence of rarely occurring fragments, which the model may not effectively contextualize. The performance difference between the one-phase and two-phase pre-training strategies is also observed.

Apart from the outcomes at the 1000 frequency level, the two-phase approach invariably results in a slightly higher loss and improved accuracy. This may be due to the difficulties in contextualizing a hybrid sequence input that starts with SMILES tokenization and subsequently employs hybrid-fragment tokenization. In contrast, the one-phase strategy teaches the model to contextualize both fragments and SMILES simultaneously from the start. Hence, two-phase models might have to recalibrate their SMILES contextualization alongside integrating fragment information, which can result in a diminished overall performance compared to the one-phase pre-training approach. It is important to note that the comparison of pre-training performance across different cutoffs and pure SMILES, as shown by the testing loss and accuracy in Table 3, is biased, as the varying sizes of the vocabularies indicate differing degrees of challenge.

The average pre-training curves of hybrid tokenization models on the testing set are illustrated in Fig. 4, separated by one-phase and two-phase. The results show a rapid improvement in the first epoch, which is characterized by a steep learning curve. This is followed by a more gradual progression in subsequent epochs, with less improvement but still evident. While the SMILES performance pattern is not illustrated, it mirrors the hybrid models with a significant initial improvement, although demonstrating lower loss values and higher accuracy rates at the end of training. The observed trends raise questions about the optimal configuration of the learning rate. Rapid early improvements hint at a robust initial grasp of data representation learning, however the plateau in later stages implies a potential overfitting or inability to further generalize from the training data. Adjusting the learning rate could help the model learn more effectively throughout the pre-training process, however we did not do so due to the high computational demands of the pre-training phase, in conjunction with the need to tune the learning rate according to each fragment frequency cutoff.

Furthermore, the consistent, although marginal, gains after the first epoch suggest that the models are still extracting valuable information at a reduced rate. This could imply that the models are approaching their capacity for learning from the given dataset, or that the complexity of the data requires more nuanced learning strategies. Further improvements to modelling could be found from an increased, and complex dataset of molecules, altering the masking rates of the masked language modelling strategy, or employing different learning strategy than masked language modelling.

In summary, in this section, we investigated the performance of our HFST strategy in masked model pre-training, finding it to be a challenging task as more infrequent fragments are included in the vocabulary. In the context of the testing loss metric, it is observed that the performance marginally declines with the application of two-phase pre-training compared to a single phase approach. This reduction in performance may be attributed to the necessity of recontextualizing the embeddings upon transitioning from the initial to the subsequent phase. Unlike two-phase, the one-phase approach employs our proposed HFST method from the outset, thereby averting the need for recontextualization. While the one-phase approach demonstrates a preferable performance compared to the two-phase approach during pre-training, we evaluate the efficacy of our proposed method for ADMET prediction in the subsequent section.

### Fine-tuning for ADMET prediction

**Fig. 5:**
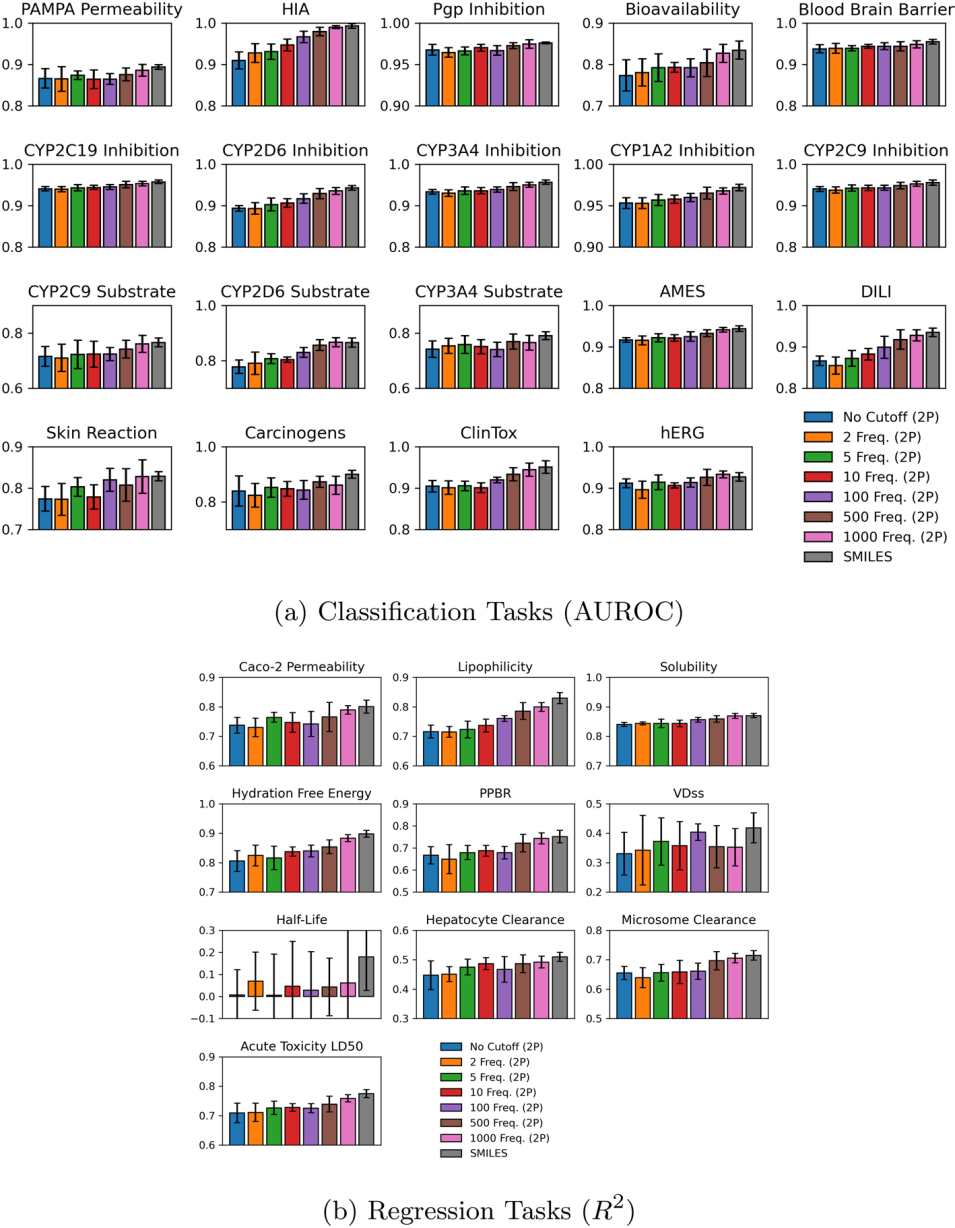
Comparison between two-phase fine-tuning experimentation and SMILES for **a** classification and **b** regression tasks, averaged among folds and executions

**Fig. 6:**
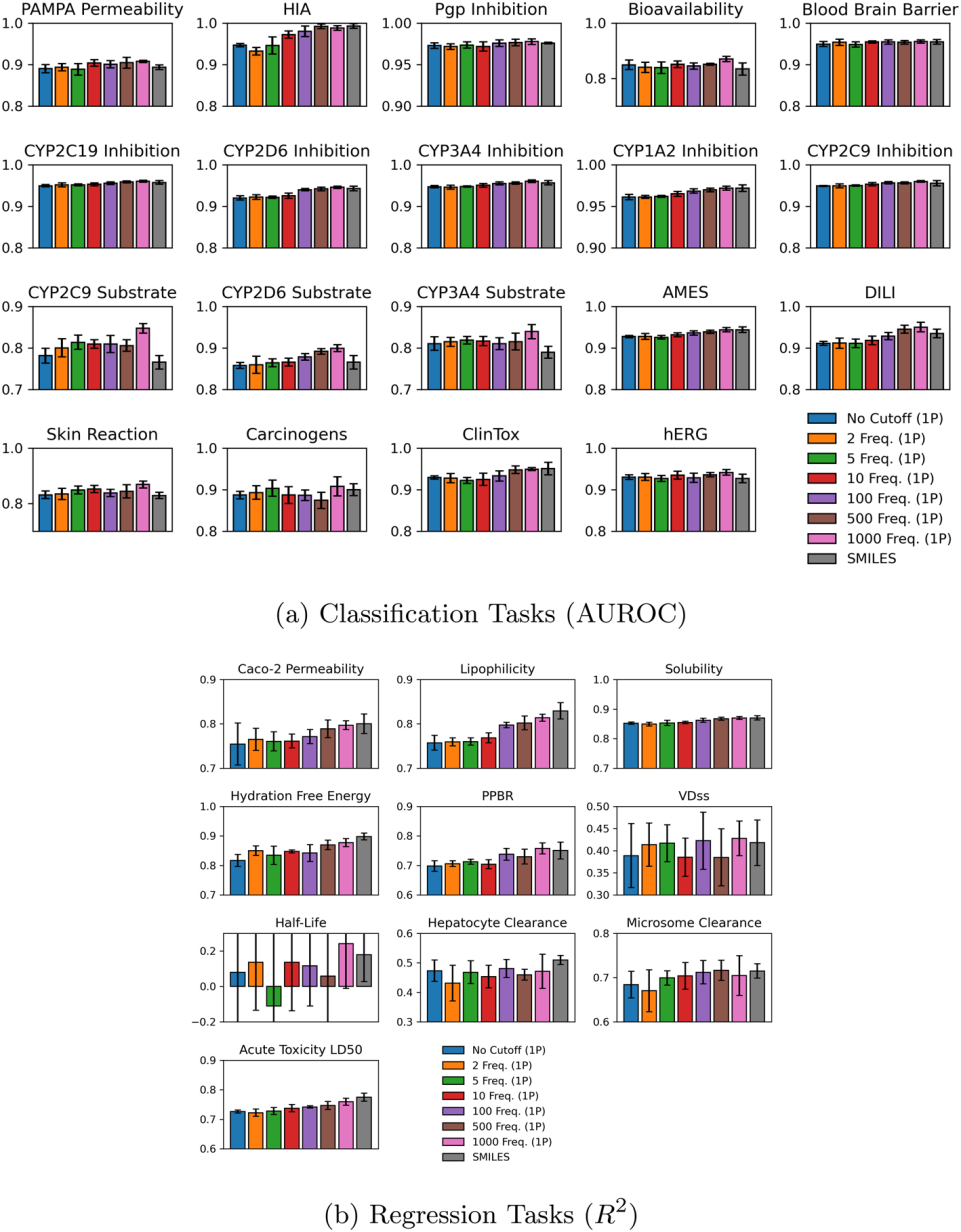
Comparison between one-phase fine-tuning experimentation and SMILES for **a** classification and **b** regression tasks, averaged among folds and executions

The performance of the hybrid and SMILES tokenization models during the final testing epoch, averaged across multiple runs and folds, is presented in Figs. 5 and 6. Additionally, Tables 4 and 5 state the resultant values, categorized by the two-phase and one-phase strategies.

In the two-phase approach, we observed that SMILES tokenization consistently achieved the best performance, followed closely by the 1000 frequency hybrid tokenization, with worse metric values as more infrequent fragments are incorporated. With rare fragments included, the model fails to effectively contextualize rare fragments and accurately predict ADMET properties. Interestingly, the 1000 frequency hybrid approach outperformed SMILES specifically in CYP2D6 substrate classification and hERG regression tasks, both of which are critical for drug metabolism and safety within the cardiovascular system.

When using one-phase pre-training, both SMILES and 1000 frequency hybrid tokenizations emerge as top performers, with a trend of lower performance for lower frequency cutoffs persisting between the one-phase and two-phase strategies. Notably, the one-phase 1000 frequency hybrid tokenization consistently outperforms SMILES across most tasks, with an exception in the blood-brain barrier predictive task, where 10 frequency matched SMILES and 1000 frequency hybrid. As well, 500 frequency hybrid outpaces all remaining frequencies and SMILES for microsome clearance prediction. This suggests that specific tasks benefit from varying tokenization strategies, as certain molecular substructures carry high predictive weight.

Overall, our models demonstrated better performance on classification tasks than on regression tasks. The binary nature of the ADMET classification tasks makes them inherently easier to predict than regression tasks, which take on continuous values. Both the hybrid and SMILES tokenization model exhibited poor performance on the half-life dataset, with suboptimal average prediction and a high standard deviation, as seen in Figs. 5 and 6. This dataset likely poses unique challenges due to the complex interplay of molecular features that affect drug half-life, some of which may not be directly related to the molecule itself. Despite the poor performance overall, one-phase 1000 frequency hybrid tokenization still outperformed SMILES on the half-life task, suggesting that the hybrid approach offers resilience in challenging predictive tasks.

### Fine-tuning on therapeutics data commons ADMET benchmark

We fine-tune our HFST model and SMILES model utilizing the ADMET group benchmark from TDC, encompassing a total of 22 datasets. The mean and standard deviation test set performance of the 1000 frequency one-phase HFST and SMILES tokenization models is presented in Table 6, provided with the corresponding performance metrics as explained in Sect. 4.5, and highlighting of best metric values. Furthermore, we provide a comparative analysis between our model and five non-ensemble models that are prominently featured on the leaderboard, having submitted entries across all benchmark datasets. This comparison spans a diverse array of machine learning methodologies, ranging from conventional ADMET prediction employing molecular fingerprints and multilayer perceptron (MLP) models to contemporary deep learning methods, including convolutional neural networks (CNNs) and GNNs. The models used for comparison include Basic ML [72], DeepPurpose (with variants Morgan + MLP and CNN, each executed separately) [73], Chemprop (a message passing GNN model) [74], and AttentiveFP (a GAT model) [54].

The results in Table 6 reveal HFST demonstrating superior performance over the traditional SMILES notation for molecular language modelling in a majority of the benchmark tasks, as corroborated by the findings in Sect. 5.2. Notably, the one-phase 1000 frequency HFST excels in predicting bioavailability and hepatocyte clearance, while SMILES tokenization shows its strengths in CYP 2C9 substrate and microsome clearance tasks. This performance underscores the potential of HFST in certain ADMET applications. Extending beyond our approach, no single model dominates across all 22 benchmark datasets, suggesting that a tailored approach of selecting specific models for specific tasks may yield the most effective strategy in predictive modeling for drug discovery. This observation aligns with the current absence of language models on the TDC leaderboard and the prevalence of GNNs, indicating a need for enhancements in training Transformer models to establish their competitive edge in this domain. The results collectively signal an opportunity for the development of more robust models capable of consistent performance across a diverse array of ADMET prediction tasks.

## Conclusion

In this study, we explore the impact of a novel hybrid fragment-SMILES tokenization (HFST) procedure alongside two pre-training strategies for Transformer-based ADMET prediction, while experimenting with a spectrum of fragment vocabularies. Our findings underscore the critical role of data representation and learning methodology in achieving accurate predictions for classification and regression tasks. Although SMILES tokenization remains a robust baseline, our hybrid approach, especially at the 1000 frequency level, consistently outperforms SMILES tokenization, under both a collection of 29 ADMET datasets and the TDC ADMET group benchmark. However, it is important to recognize that the selection of a frequency cutoff significantly impacts model performance, and incorporating lower frequency fragments tends to have a detrimental affect on ADMET predictions. Therefore, adjusting the fragments frequency emerges as an important hyperparameter that requires tuning before model training.

From our experimentation, the need for learning rate optimization is clear, and further tuning could yield substantial improvements in ADMET prediction accuracy. Due to the large computational cost of each experiment, and the number of experiments performed in this study, we did not tune the learning rate. However, we predict that for each differing frequency cutoff vocabulary, the learning rate must be tuned. In addition, we propose adjusting the masked language modelling approach to prioritize converting fragments into mask tokens and assigning them a higher weight during loss calculation. By doing so, the model should more effectively contextualize between fragments and SMILES tokens within our hybrid approach. Given the limited efficiency of the masked language modelling strategy, where only 15% of tokens are used for prediction, we recommend exploring an encoder-decoder Transformer model and language modelling strategy. A full Transformer model learns to contextualize entire sequences at once, potentially addressing some of the limitations observed in our current approach. Last but not the least, the hybrid encoding idea is applicable to other line notation representation methods for molecules, such as SELFIES [10], along with suitable fragmentation techniques, and to a range of quantitative structure activity relationship (QSAR) predictive tasks. This generalization needs to be comprehensively studied as future work.

## Data Availability

The molecular data used in this research is a combined set of the MOSES, ChEMBL, and ZINC-250K databases for pre-training, 29 ADMET datasets for fine-tuning, and benchmark datasets under the ADMET group leaderboard, all of which have been gathered from Therapeutics Data Commons (accessible via https://tdcommons.ai). The implementation of this research can be found at https://github.com/Pixelatory/HybridFragmentTokenization.

## Appendix A: Comparison of ADMET modelling experimentation

The detailed results for the two-phase and one-phase strategies pre-training and then fine-tuning on ADMET tasks are given in Tables 4 and 5.

**Table 4:** Comparison of ADMET modelling experimentation using two-phase pre-training

Prediction Task	SMILES	No Cutoff	2 Freq.	5 Freq.	10 Freq.	100 Freq.	500 Freq.	1000 Freq.
Caco-2	$\mathbf {0.8 \pm 0.022}$	$0.737 \pm 0.027$	$0.73 \pm 0.031$	$0.764 \pm 0.017$	$0.747 \pm 0.033$	$0.742 \pm 0.043$	$0.765 \pm 0.05$	$0.789 \pm 0.015$
PAMPA	$\mathbf {0.894 \pm 0.006}$	$0.866 \pm 0.023$	$0.865 \pm 0.03$	$0.874 \pm 0.01$	$0.864 \pm 0.023$	$0.865 \pm 0.013$	$0.876 \pm 0.015$	$0.886 \pm 0.014$
HIA	$\mathbf {0.993 \pm 0.006}$	$0.91 \pm 0.021$	$0.928 \pm 0.023$	$0.931 \pm 0.019$	$0.947 \pm 0.014$	$0.966 \pm 0.014$	$0.979 \pm 0.01$	$0.99 \pm 0.003$
Pgp Inhibition	$\mathbf {0.976 \pm 0.001}$	$0.968 \pm 0.007$	$0.965 \pm 0.006$	$0.966 \pm 0.005$	$0.97 \pm 0.004$	$0.967 \pm 0.006$	$0.973 \pm 0.004$	$0.975 \pm 0.005$
Bioavailability	$\mathbf {0.835 \pm 0.022}$	$0.774 \pm 0.037$	$0.781 \pm 0.033$	$0.792 \pm 0.033$	$0.793 \pm 0.012$	$0.792 \pm 0.022$	$0.804 \pm 0.033$	$0.827 \pm 0.022$
Lipophilicity	$\mathbf {0.829 \pm 0.019}$	$0.716 \pm 0.021$	$0.715 \pm 0.018$	$0.723 \pm 0.029$	$0.736 \pm 0.022$	$0.76 \pm 0.01$	$0.785 \pm 0.029$	$0.799 \pm 0.015$
Solubility	$\mathbf {0.87 \pm 0.007}$	$0.84 \pm 0.006$	$0.844 \pm 0.005$	$0.843 \pm 0.015$	$0.843 \pm 0.011$	$0.856 \pm 0.008$	$0.859 \pm 0.011$	$0.869 \pm 0.008$
FreeSolv	$\mathbf {0.898 \pm 0.012}$	$0.806 \pm 0.035$	$0.824 \pm 0.035$	$0.816 \pm 0.04$	$0.838 \pm 0.016$	$0.84 \pm 0.02$	$0.854 \pm 0.024$	$0.883 \pm 0.012$
BBB	$\mathbf {0.955 \pm 0.005}$	$0.938 \pm 0.01$	$0.939 \pm 0.012$	$0.939 \pm 0.006$	$0.944 \pm 0.005$	$0.944 \pm 0.008$	$0.944 \pm 0.011$	$0.949 \pm 0.009$
PPBR	$\mathbf {0.751 \pm 0.028}$	$0.667 \pm 0.039$	$0.649 \pm 0.066$	$0.679 \pm 0.032$	$0.687 \pm 0.025$	$0.679 \pm 0.029$	$0.722 \pm 0.039$	$0.743 \pm 0.025$
VDss	$\mathbf {0.418 \pm 0.051}$	$0.33 \pm 0.072$	$0.342 \pm 0.118$	$0.372 \pm 0.081$	$0.358 \pm 0.082$	$0.404 \pm 0.028$	$0.354 \pm 0.072$	$0.353 \pm 0.063$
CYP 2C19	$\mathbf {0.958 \pm 0.005}$	$0.941 \pm 0.005$	$0.94 \pm 0.006$	$0.943 \pm 0.008$	$0.944 \pm 0.005$	$0.945 \pm 0.006$	$0.951 \pm 0.008$	$0.953 \pm 0.005$
CYP 2D6	$\mathbf {0.943 \pm 0.005}$	$0.893 \pm 0.007$	$0.893 \pm 0.014$	$0.903 \pm 0.015$	$0.906 \pm 0.011$	$0.917 \pm 0.011$	$0.929 \pm 0.012$	$0.936 \pm 0.008$
CYP 3A4	$\mathbf {0.956 \pm 0.005}$	$0.934 \pm 0.005$	$0.93 \pm 0.008$	$0.936 \pm 0.009$	$0.936 \pm 0.008$	$0.939 \pm 0.006$	$0.946 \pm 0.009$	$0.95 \pm 0.006$
CYP 1A2	$\mathbf {0.972 \pm 0.004}$	$0.953 \pm 0.007$	$0.953 \pm 0.007$	$0.957 \pm 0.007$	$0.958 \pm 0.005$	$0.96 \pm 0.005$	$0.965 \pm 0.007$	$0.968 \pm 0.004$
CYP 2C9	$\mathbf {0.956 \pm 0.006}$	$0.94 \pm 0.006$	$0.937 \pm 0.008$	$0.942 \pm 0.008$	$0.943 \pm 0.006$	$0.943 \pm 0.006$	$0.948 \pm 0.008$	$0.953 \pm 0.006$
CYP2C9 Sub.	$\mathbf {0.766 \pm 0.016}$	$0.715 \pm 0.036$	$0.71 \pm 0.049$	$0.722 \pm 0.051$	$0.724 \pm 0.047$	$0.723 \pm 0.024$	$0.741 \pm 0.032$	$0.76 \pm 0.031$
CYP2D6 Sub.	$0.866 \pm 0.016$	$0.778 \pm 0.024$	$0.79 \pm 0.04$	$0.807 \pm 0.018$	$0.804 \pm 0.009$	$0.83 \pm 0.018$	$0.856 \pm 0.019$	$\mathbf {0.867 \pm 0.017}$
CYP3A4 Sub.	$\mathbf {0.79 \pm 0.014}$	$0.742 \pm 0.03$	$0.754 \pm 0.028$	$0.759 \pm 0.032$	$0.751 \pm 0.025$	$0.74 \pm 0.026$	$0.769 \pm 0.027$	$0.765 \pm 0.026$
Half Life	$\mathbf {0.179 \pm 0.151}$	$0.007 \pm 0.115$	$0.07 \pm 0.131$	$0.006 \pm 0.187$	$0.047 \pm 0.203$	$0.029 \pm 0.174$	$0.043 \pm 0.13$	$0.061 \pm 0.256$
Hepatocyte Clear.	$\mathbf {0.51 \pm 0.015}$	$0.447 \pm 0.049$	$0.451 \pm 0.025$	$0.475 \pm 0.027$	$0.487 \pm 0.02$	$0.467 \pm 0.043$	$0.486 \pm 0.03$	$0.492 \pm 0.02$
Microsome Clear.	$\mathbf {0.715 \pm 0.016}$	$0.655 \pm 0.023$	$0.639 \pm 0.034$	$0.656 \pm 0.029$	$0.658 \pm 0.039$	$0.661 \pm 0.028$	$0.697 \pm 0.031$	$0.705 \pm 0.016$
LD50	$\mathbf {0.774 \pm 0.014}$	$0.709 \pm 0.033$	$0.711 \pm 0.031$	$0.726 \pm 0.022$	$0.728 \pm 0.013$	$0.725 \pm 0.015$	$0.739 \pm 0.027$	$0.759 \pm 0.012$
hERG	$0.927 \pm 0.01$	$0.912 \pm 0.011$	$0.896 \pm 0.021$	$0.914 \pm 0.018$	$0.906 \pm 0.006$	$0.913 \pm 0.011$	$0.926 \pm 0.019$	$\mathbf {0.933 \pm 0.008}$
AMES	$\mathbf {0.944 \pm 0.007}$	$0.917 \pm 0.006$	$0.915 \pm 0.01$	$0.922 \pm 0.01$	$0.921 \pm 0.008$	$0.925 \pm 0.011$	$0.933 \pm 0.009$	$0.941 \pm 0.005$
DILI	$\mathbf {0.935 \pm 0.01}$	$0.866 \pm 0.012$	$0.855 \pm 0.021$	$0.872 \pm 0.019$	$0.882 \pm 0.014$	$0.899 \pm 0.026$	$0.918 \pm 0.024$	$0.927 \pm 0.013$
Skin Reaction	$\mathbf {0.829 \pm 0.011}$	$0.774 \pm 0.03$	$0.773 \pm 0.038$	$0.803 \pm 0.023$	$0.779 \pm 0.029$	$0.819 \pm 0.028$	$0.807 \pm 0.039$	$0.828 \pm 0.04$
Carcinogens	$\mathbf {0.9 \pm 0.014}$	$0.839 \pm 0.054$	$0.824 \pm 0.043$	$0.852 \pm 0.035$	$0.847 \pm 0.027$	$0.843 \pm 0.034$	$0.872 \pm 0.02$	$0.86 \pm 0.033$
ClinTox	$\mathbf {0.951 \pm 0.015}$	$0.904 \pm 0.014$	$0.901 \pm 0.016$	$0.905 \pm 0.011$	$0.901 \pm 0.012$	$0.919 \pm 0.007$	$0.933 \pm 0.016$	$0.944 \pm 0.015$

Entries in boldface highlight the best mean performance in individual tasks

**Table 5:** Comparison of ADMET modelling experimentation using one-phase pre-training﻿

Task	SMILES	No Cutoff	2 Freq.	5 Freq.	10 Freq.	100 Freq.	500 Freq.	1000 Freq.
Caco-2	$\mathbf {0.8 \pm 0.022}$	$0.754 \pm 0.047$	$0.765 \pm 0.025$	$0.761 \pm 0.022$	$0.761 \pm 0.016$	$0.771 \pm 0.016$	$0.789 \pm 0.02$	$0.797 \pm 0.01$
PAMPA	$0.894 \pm 0.006$	$0.89 \pm 0.01$	$0.894 \pm 0.009$	$0.889 \pm 0.014$	$0.904 \pm 0.008$	$0.901 \pm 0.008$	$0.905 \pm 0.013$	$\mathbf {0.908 \pm 0.003}$
HIA	$\mathbf {0.993 \pm 0.006}$	$0.947 \pm 0.004$	$0.932 \pm 0.009$	$0.946 \pm 0.02$	$0.972 \pm 0.009$	$0.98 \pm 0.012$	$0.992 \pm 0.006$	$0.988 \pm 0.006$
Pgp Inhibition	$0.976 \pm 0.001$	$0.973 \pm 0.003$	$0.972 \pm 0.003$	$0.974 \pm 0.004$	$0.972 \pm 0.006$	$0.976 \pm 0.004$	$0.977 \pm 0.004$	$\mathbf {0.978 \pm 0.003}$
Bioavailability	$0.835 \pm 0.022$	$0.85 \pm 0.017$	$0.84 \pm 0.018$	$0.839 \pm 0.021$	$0.852 \pm 0.012$	$0.845 \pm 0.011$	$0.851 \pm 0.003$	$\mathbf {0.87 \pm 0.01}$
Lipophilicity	$\mathbf {0.829 \pm 0.019}$	$0.757 \pm 0.017$	$0.759 \pm 0.009$	$0.76 \pm 0.008$	$0.768 \pm 0.011$	$0.797 \pm 0.006$	$0.802 \pm 0.015$	$0.814 \pm 0.008$
Solubility	$\mathbf {0.87 \pm 0.007}$	$0.852 \pm 0.004$	$0.849 \pm 0.006$	$0.853 \pm 0.009$	$0.855 \pm 0.004$	$0.862 \pm 0.007$	$0.867 \pm 0.005$	$\mathbf {0.87 \pm 0.005}$
FreeSolv	$\mathbf {0.898 \pm 0.012}$	$0.817 \pm 0.02$	$0.85 \pm 0.016$	$0.835 \pm 0.031$	$0.848 \pm 0.005$	$0.842 \pm 0.028$	$0.869 \pm 0.016$	$0.877 \pm 0.013$
BBB	$\mathbf {0.955 \pm 0.005}$	$0.95 \pm 0.006$	$0.954 \pm 0.007$	$0.949 \pm 0.006$	$\mathbf {0.955 \pm 0.003}$	$0.954 \pm 0.005$	$0.954 \pm 0.004$	$\mathbf {0.955 \pm 0.004}$
PPBR	$0.751 \pm 0.028$	$0.698 \pm 0.018$	$0.706 \pm 0.01$	$0.713 \pm 0.009$	$0.704 \pm 0.016$	$0.738 \pm 0.02$	$0.73 \pm 0.025$	$\mathbf {0.758 \pm 0.019}$
VDss	$0.418 \pm 0.051$	$0.389 \pm 0.072$	$0.414 \pm 0.049$	$0.417 \pm 0.042$	$0.385 \pm 0.043$	$0.423 \pm 0.064$	$0.385 \pm 0.064$	$\mathbf {0.428 \pm 0.039}$
CYP 2C19	$0.958 \pm 0.005$	$0.95 \pm 0.003$	$0.951 \pm 0.005$	$0.951 \pm 0.002$	$0.953 \pm 0.004$	$0.955 \pm 0.003$	$0.959 \pm 0.003$	$\mathbf {0.96 \pm 0.002}$
CYP 2D6	$0.943 \pm 0.005$	$0.92 \pm 0.005$	$0.922 \pm 0.006$	$0.922 \pm 0.003$	$0.925 \pm 0.006$	$0.94 \pm 0.003$	$0.942 \pm 0.004$	$\mathbf {0.946 \pm 0.003}$
CYP 3A4	$0.956 \pm 0.005$	$0.947 \pm 0.003$	$0.946 \pm 0.005$	$0.948 \pm 0.002$	$0.951 \pm 0.005$	$0.955 \pm 0.004$	$0.956 \pm 0.003$	$\mathbf {0.96 \pm 0.003}$
CYP 1A2	$\mathbf {0.972 \pm 0.004}$	$0.961 \pm 0.003$	$0.961 \pm 0.002$	$0.962 \pm 0.001$	$0.965 \pm 0.003$	$0.968 \pm 0.002$	$0.97 \pm 0.002$	$\mathbf {0.972 \pm 0.002}$
CYP 2C9	$0.956 \pm 0.006$	$0.949 \pm 0.001$	$0.949 \pm 0.005$	$0.95 \pm 0.002$	$0.953 \pm 0.004$	$0.957 \pm 0.003$	$0.956 \pm 0.003$	$\mathbf {0.96 \pm 0.002}$
CYP2C9 Sub.	$0.766 \pm 0.016$	$0.782 \pm 0.018$	$0.8 \pm 0.022$	$0.814 \pm 0.017$	$0.81 \pm 0.01$	$0.81 \pm 0.021$	$0.806 \pm 0.014$	$\mathbf {0.847 \pm 0.011}$
CYP2D6 Sub.	$0.866 \pm 0.016$	$0.858 \pm 0.007$	$0.86 \pm 0.021$	$0.864 \pm 0.01$	$0.866 \pm 0.01$	$0.878 \pm 0.008$	$0.892 \pm 0.006$	$\mathbf {0.899 \pm 0.008}$
CYP3A4 Sub.	$0.79 \pm 0.014$	$0.811 \pm 0.016$	$0.815 \pm 0.011$	$0.819 \pm 0.009$	$0.817 \pm 0.011$	$0.81 \pm 0.014$	$0.815 \pm 0.021$	$\mathbf {0.84 \pm 0.017}$
Half Life	$0.179 \pm 0.151$	$0.08 \pm 0.337$	$0.137 \pm 0.271$	$-0.11 \pm 0.578$	$0.136 \pm 0.273$	$0.117 \pm 0.227$	$0.059 \pm 0.338$	$\mathbf {0.242 \pm 0.252}$
Hepatocyte Clear.	$\mathbf {0.51 \pm 0.015}$	$0.473 \pm 0.036$	$0.431 \pm 0.06$	$0.468 \pm 0.039$	$0.453 \pm 0.038$	$0.48 \pm 0.031$	$0.46 \pm 0.018$	$0.472 \pm 0.058$
Microsome Clear.	$0.715 \pm 0.016$	$0.684 \pm 0.03$	$0.67 \pm 0.047$	$0.699 \pm 0.016$	$0.704 \pm 0.03$	$0.712 \pm 0.026$	$\mathbf {0.716 \pm 0.023}$	$0.705 \pm 0.045$
LD50	$\mathbf {0.774 \pm 0.014}$	$0.726 \pm 0.005$	$0.722 \pm 0.012$	$0.728 \pm 0.012$	$0.737 \pm 0.012$	$0.741 \pm 0.004$	$0.746 \pm 0.014$	$0.759 \pm 0.012$
hERG	$0.927 \pm 0.01$	$0.93 \pm 0.006$	$0.93 \pm 0.008$	$0.926 \pm 0.007$	$0.935 \pm 0.01$	$0.928 \pm 0.011$	$0.936 \pm 0.005$	$\mathbf {0.941 \pm 0.007}$
AMES	$\mathbf {0.944 \pm 0.007}$	$0.927 \pm 0.003$	$0.927 \pm 0.007$	$0.926 \pm 0.004$	$0.932 \pm 0.004$	$0.936 \pm 0.005$	$0.939 \pm 0.004$	$\mathbf {0.944 \pm 0.005}$
DILI	$0.935 \pm 0.01$	$0.911 \pm 0.005$	$0.912 \pm 0.012$	$0.911 \pm 0.01$	$0.918 \pm 0.01$	$0.929 \pm 0.008$	$0.945 \pm 0.009$	$\mathbf {0.95 \pm 0.011}$
Skin Reaction	$0.829 \pm 0.011$	$0.831 \pm 0.013$	$0.835 \pm 0.02$	$0.849 \pm 0.014$	$0.852 \pm 0.013$	$0.838 \pm 0.013$	$0.844 \pm 0.024$	$\mathbf {0.868 \pm 0.012}$
Carcinogens	$0.9 \pm 0.014$	$0.887 \pm 0.009$	$0.893 \pm 0.016$	$0.903 \pm 0.019$	$0.887 \pm 0.02$	$0.886 \pm 0.012$	$0.874 \pm 0.019$	$\mathbf {0.908 \pm 0.023}$
ClinTox	$\mathbf {0.951 \pm 0.015}$	$0.929 \pm 0.004$	$0.928 \pm 0.011$	$0.922 \pm 0.007$	$0.925 \pm 0.015$	$0.933 \pm 0.013$	$0.948 \pm 0.009$	$0.949 \pm 0.003$

Entries in boldface highlight the best mean performance in individual tasks

**Table 6:** Comparison of ADMET prediction models on TDC ADMET group benchmark﻿

Task	1000 Freq.	SMILES	Basic ML	Morgan + MLP	CNN (DeepPurpose)	Chemprop	AttentiveFP
Caco-2 (MAE)	$0.508 \pm 0.041$	$0.605 \pm 0.081$	$\mathbf {0.321 \pm 0.005}$	$0.908 \pm 0.060$	$0.446 \pm 0.036$	$0.344 \pm 0.015$	$0.401 \pm 0.032$
HIA (AUROC)	$0.832 \pm 0.031$	$0.808 \pm 0.071$	$0.818 \pm 0.010$	$0.807 \pm 0.072$	$0.869 \pm 0.026$	$0.965 \pm 0.005$	$\mathbf {0.974 \pm 0.007}$
Pgp Inhibition (AUROC)	$0.864 \pm 0.011$	$0.870 \pm 0.018$	$0.818 \pm 0.000$	$0.880 \pm 0.006$	$\mathbf {0.908 \pm 0.012}$	$0.860 \pm 0.036$	$0.892 \pm 0.012$
Bioavailability (AUROC)	$\mathbf {0.645 \pm 0.034}$	$0.609 \pm 0.076$	$0.523 \pm 0.011$	$0.581 \pm 0.086$	$0.613 \pm 0.013$	$0.581 \pm 0.024$	$0.632 \pm 0.039$
Lipophilicity (MAE)	$0.847 \pm 0.022$	$0.867 \pm 0.023$	$0.617 \pm 0.003$	$0.701 \pm 0.009$	$0.743 \pm 0.020$	$\mathbf {0.470 \pm 0.009}$	$0.572 \pm 0.007$
Solubility (MAE)	$1.167 \pm 0.029$	$1.176 \pm 0.038$	$0.828 \pm 0.002$	$1.203 \pm 0.019$	$1.023 \pm 0.023$	$0.829 \pm 0.022$	$\mathbf {0.776 \pm 0.008}$
BBB (AUROC)	$0.802 \pm 0.029$	$0.769 \pm 0.037$	$0.811 \pm 0.013$	$0.823 \pm 0.015$	$0.781 \pm 0.030$	$0.821 \pm 0.112$	$\mathbf {0.855 \pm 0.011}$
PPBR (MAE)	$9.804 \pm 0.591$	$9.638 \pm 1.014$	$9.185 \pm 0.000$	$12.848 \pm 0.362$	$11.106 \pm 0.358$	$\mathbf {7.788 \pm 0.210}$	$9.373 \pm 0.335$
VDss (Spearman)	$0.399 \pm 0.044$	$0.381 \pm 0.101$	$\mathbf {0.627 \pm 0.010}$	$0.493 \pm 0.011$	$0.226 \pm 0.114$	$0.491 \pm 0.046$	$0.241 \pm 0.145$
CYP 2D6 (AUPRC)	$0.395 \pm 0.027$	$0.373 \pm 0.049$	$0.358 \pm 0.000$	$0.587 \pm 0.011$	$0.544 \pm 0.053$	$\mathbf {0.649 \pm 0.016}$	$0.646 \pm 0.014$
CYP 3A4 (AUPRC)	$0.665 \pm 0.034$	$0.666 \pm 0.014$	$0.654 \pm 0.000$	$0.827 \pm 0.009$	$0.821 \pm 0.003$	$\mathbf {0.862 \pm 0.003}$	$0.851 \pm 0.006$
CYP 2C9 (AUPRC)	$0.635 \pm 0.012$	$0.620 \pm 0.016$	$0.556 \pm 0.000$	$0.715 \pm 0.004$	$0.713 \pm 0.006$	$\mathbf {0.754 \pm 0.002}$	$0.749 \pm 0.004$
CYP2C9 Sub. (AUPRC)	$0.381 \pm 0.053$	$\mathbf {0.388 \pm 0.052}$	$0.281 \pm 0.000$	$0.380 \pm 0.015$	$0.367 \pm 0.059$	$0.382 \pm 0.019$	$0.375 \pm 0.032$
CYP2D6 Sub. (AUPRC)	$0.525 \pm 0.057$	$0.501 \pm 0.072$	$0.478 \pm 0.018$	$\mathbf {0.671 \pm 0.066}$	$0.485 \pm 0.037$	$0.632 \pm 0.037$	$0.574 \pm 0.030$
CYP3A4 Sub. (AUROC)	$0.593 \pm 0.037$	$0.571 \pm 0.032$	$0.605 \pm 0.000$	$0.633 \pm 0.013$	$\mathbf {0.662 \pm 0.031}$	$0.596 \pm 0.018$	$0.576 \pm 0.025$
Half Life (Spearman)	$0.272 \pm 0.053$	$0.232 \pm 0.072$	$\mathbf {0.438 \pm 0.011}$	$0.329 \pm 0.083$	$0.038 \pm 0.138$	$0.265 \pm 0.032$	$0.085 \pm 0.068$
Hepatocyte Clear. (Spearman)	$\mathbf {0.441 \pm 0.049}$	$0.431 \pm 0.044$	$0.440 \pm 0.003$	$0.272 \pm 0.068$	$0.235 \pm 0.021$	$0.431 \pm 0.006$	$0.289 \pm 0.022$
Microsome Clear. (Spearman)	$0.557 \pm 0.042$	$\mathbf {0.585 \pm 0.032}$	$0.518 \pm 0.005$	$0.492 \pm 0.020$	$0.252 \pm 0.116$	$0.555 \pm 0.022$	$0.365 \pm 0.055$
LD50 (MAE)	$0.763 \pm 0.021$	$0.781 \pm 0.025$	$0.636 \pm 0.001$	$0.649 \pm 0.019$	$0.675 \pm 0.011$	$\mathbf {0.606 \pm 0.024}$	$0.678 \pm 0.012$
hERG (AUROC)	$0.728 \pm 0.031$	$0.713 \pm 0.040$	$0.715 \pm 0.011$	$0.736 \pm 0.023$	$0.754 \pm 0.037$	$0.721 \pm 0.045$	$\mathbf {0.825 \pm 0.007}$
AMES (AUROC)	$0.697 \pm 0.022$	$0.656 \pm 0.014$	$0.716 \pm 0.000$	$0.794 \pm 0.008$	$0.776 \pm 0.015$	$\mathbf {0.842 \pm 0.014}$	$0.814 \pm 0.008$
DILI (AUROC)	$0.780 \pm 0.050$	$0.777 \pm 0.011$	$0.700 \pm 0.000$	$0.832 \pm 0.021$	$0.792 \pm 0.016$	$\mathbf {0.899 \pm 0.008}$	$0.886 \pm 0.015$

Entries in boldface highlight the best mean performance in individual tasks

## Abbreviations

2D: Two-dimensional
3D: Three-dimensional
ADMET: Absorption, distribution, metabolism, excretion, and toxicity
AI: Artificial intelligence
AUPRC: Area under the precision-recall curve
AUROC: Area under the receiver operating characteristic curve
BERT: Bidirectional encoder representations from transformers
CNN: Convolutional neural network
HFST: Hybrid-fragment SMILES tokenization
GAT: Graph attention network
GCN: Graph convolutional neural network
GIN: Graph isomorphism network
GNN: Graph-based neural network
GPT: Generative pre-trained transformer
MAE: Mean absolute error
ML: Machine learning
MLP: Multilayer perceptron
MPNN: Message passing neural network
MTL-BERT: Multi-task learning BERT
NLP: Natural language processing
QSAR: Quantitative structure activity relationship
$R^2$: Coefficient of determination
RNN: Recurrent neural network
SELFIES: SELF-referencing embedded string
SMILES: Simplified molecular input line entry system
Spearman: Spearman’s rank correlation coefficient
TDC: Therapeutics data commons
